# The refractory secondary hyperparathyroidism presenting with retro-orbital brown tumor: a case report

**DOI:** 10.1186/s12882-024-03455-8

**Published:** 2024-01-05

**Authors:** Cihan Uysal, Tugba Yilmaz, Hamiyet Ozkan, Ozlem Canoz, Bulent Tokgoz

**Affiliations:** 1 Department of Nephrology, Erciyes University School of Medicine, Dede Efendi Street, Köşk District, Kayseri, Melikgazi 38030 Turkey; 2 Department of Internal Medicine, Erciyes University School of Medicine, Kayseri, Turkey; 3 Department of Pathology, Erciyes University School of Medicine, Kayseri, Turkey

**Keywords:** Brown tumors, Orbita, Secondary hyperparathyroidism, Dialysis

## Abstract

**Background:**

Tertiary hyperparathyroidism describes the autonomous and excessive secretion of parathyroid hormone (PTH) by the parathyroid glands after longstanding secondary hyperparathyroidism in chronic kidney disease. Brown tumors are a sign of uncontrolled hyperparathyroidism. In this case, we have reported a refractory and destructive hyperparathyroidism storm. Also, it presented with atypical onset and unexpected adenoma location.

**Case presentation:**

A 37-year-old man was diagnosed with end-stage kidney disease 22 years ago. He has been undergoing dialysis treatment since that time. Recently, he was admitted to the ophthalmology department due to the unilateral anterior bulging of the right eye and drooping of the eyelid. Magnetic resonance imaging exhibited an extraconal mass lesion located in the right orbital posterior superolateral position. Computerized tomography scans considered expansile bone lesion with peripheral calcification and originating from the sphenoid wing. The bone mass lesion was resected via craniotomy due to the compressive effect. The pathological findings were consistent with brown tumors. Plasma intact PTH level was 4557 pg/mL. The patient informed that he underwent parathyroidectomy and two leg fractures operation in a medical query. Parathyroid scintigraphy determined three distinct foci consistent with adenomas and one of them was in mediastenum. Second parathyroidectomy was recommended to the patient but the patient refused surgery. Despite his medication and dialysis regimen being revised, PTH levels were maintained at higher levels in follow-up.

**Conclusions:**

We presented a hyperparathyroidism case that was resistant to all treatments and exhibited all the severe complications in a long-term dialysis patient. Furthermore, this case has revealed the importance and difficulty of secondary hyperparathyroidism management.

## Background

Parathyroid hormone (PTH) is secreted by the parathyroid glands and plays a crucial role in calcium and skeletal metabolism. Secretion of PTH is regulated essentially by serum calcium levels [1]. Calcium metabolism is a complex process involving multiple factors that tightly interact with each other.PTH acts as a stimulus for increased osteoclast activity, leading to calcium and phosphorus resorption from the bone. PTH also activates vitamin D in the kidneys. Vitamin D enhances calcium and phosphorus absorption from the gastrointestinal tract and kidney, also suppresses PTH secretion. Fibroblast growth factor 23 (FGF-23) is a phosphaturic hormoneand excreted by osteocytes. FGF-23 also inhibits vitamin D activation in kidney [2].

Hyperparathyroidism is a widespread disorder that can onset at all ages and is characterized by three forms. Primary hyperparathyroidism occurs due to excessive secretion of PTH from the parathyroid glands and is characterized by hypercalcemia and decreased serum phosphorus levels. Secondary hyperparathyroidism (SHPT) is most commonly observed in chronic kidney disease (CKD) and is characterized by hyperphosphatemia and decreased ionized calcium levels. Tertiary hyperparathyroidism describes the autonomous and excessive secretion of PTH by the parathyroid glands after longstanding SHPT [3].

SHPT is initiated by phosphate retention after a decline in glomerular filtration rate (GFR) below 50 ml/min/1.73 m². The first response is increased secretion of FGF-23. Subsequently, decreased calcitriol levels lead to hypocalcemia and triggering increased PTH secretion. The severity of SHPT increase with declining kidney function. SHPT is associated with disturbances in bone turnover and extraosseous calcifications [4]. Impaired bone remodeling and mineralization leads to secondary skeletal deformities. Chest wall deformities and kyphoscoliosis are frequently observed examples of SHPT-related deformities. However, SHPT is not limited to skeletal manifestations and also impacts cardiovascular mortality in patients with end-stage kidney disease (ESKD). SHPT causesvarious extra-skeletal symptoms like as uremic pruritus [5].

Longstanding SHPT results in osteitis fibrosa cystica (OFC), often defined as a radiological sign of renal osteodystrophy [5]. OFC is an old terminology for essentially high turnover bone disease in CKD. Hyperparathyroidism leads to cortical bone resorption, microfracture development, andbleeding in bone tissues. Multinucleated macrophages migrate to this area, leading to the ingrowth of granulation tissue. Brown tumors (BT) are rare and severe form of OFC. Histologically, BT represents localized bony accumulations of fibrous tissue and giant cells. The brown coloration is due to hemosiderin deposition. BT manifest as focal bony lesions with increased osteoclastic activity and trabecular fibrosis [6].

BT are typically observed as well-defined lesions in the skeleton, most commonly detected in the ribs, clavicle, pelvic girdle, and mandible. Despite being benign bone lesions, BT appear occasionally as an aggressive malignant bone tumors on imaging. The orbital bones unexpected location for BT and have been reported rarely. In this case, we present a long-term dialysis patient having refractory hyperparathyroidism withvigorouscomplications.

## Case presentation

A 37-year-old man has been undergoing hemodialysis (HD), three times a week, treatment for the past 15 years. He also underwent peritoneal dialysis for five years before HD. The primary etiology of the kidney disease was unknown. He was admitted to the ophthalmology clinics due to the unilateral anterior bulging of the right eye and drooping of the eyelid. This complaint was onset last month and has augmented over time.

The patient was alert and cooperative on the physical examination. The unilateral exophthalmos was observed in the right eye. Both direct and indirect pupillary light reflexes were isochoric. Limited upward and outward gaze was noted in the right eye. Papilledema and swelling of the optic disc were observed in the right eye. No abnormal neurological reflexes were detected. Any mass lesions or lymphadenopathy were not detected elsewhere in his body. Additionally, an arteriovenous fistula was visible on the left forearm. The patient did not report any accompanying symptoms, such as episodes of syncope, seizures, or fecal/urinary incontinence.

**Fig. 1:**
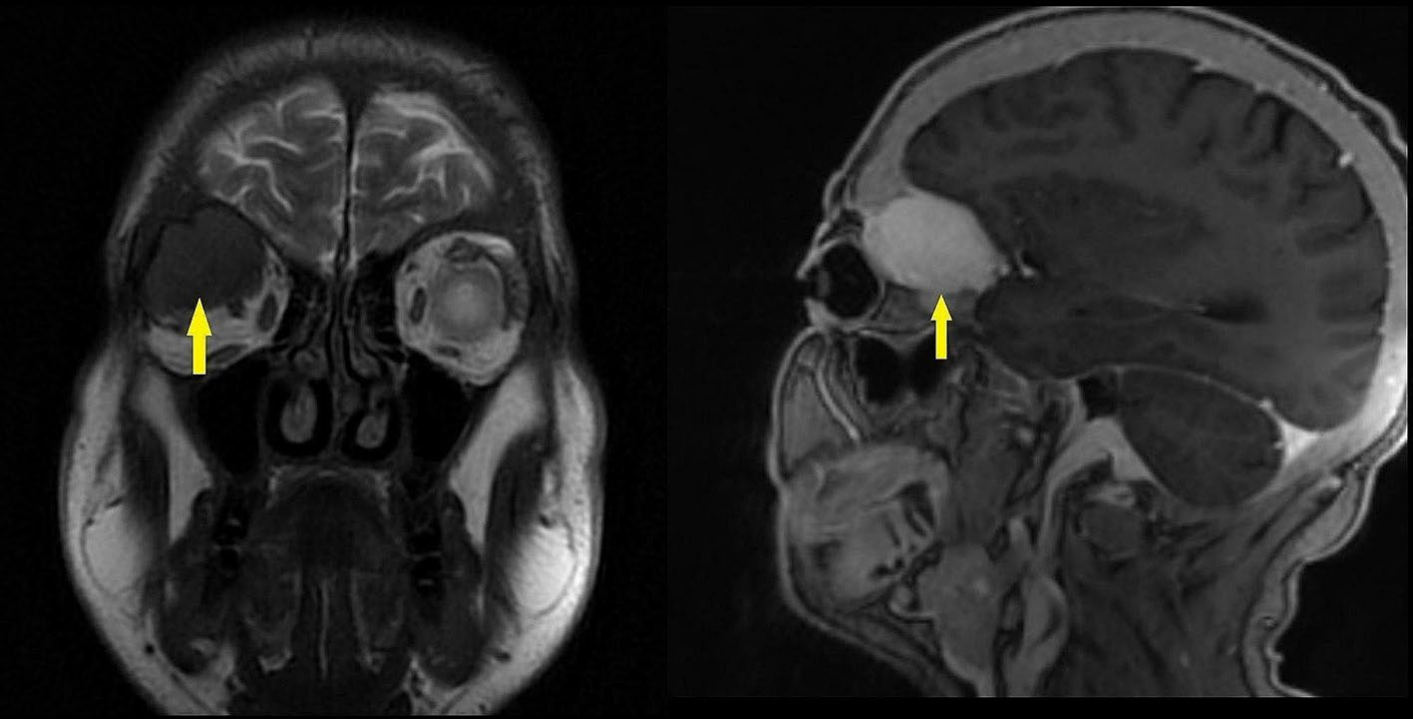
Brain MRI images. Yellow arrows point to of extraconal mass lesion in the right orbital posterior superolateral position

**Fig. 2:**
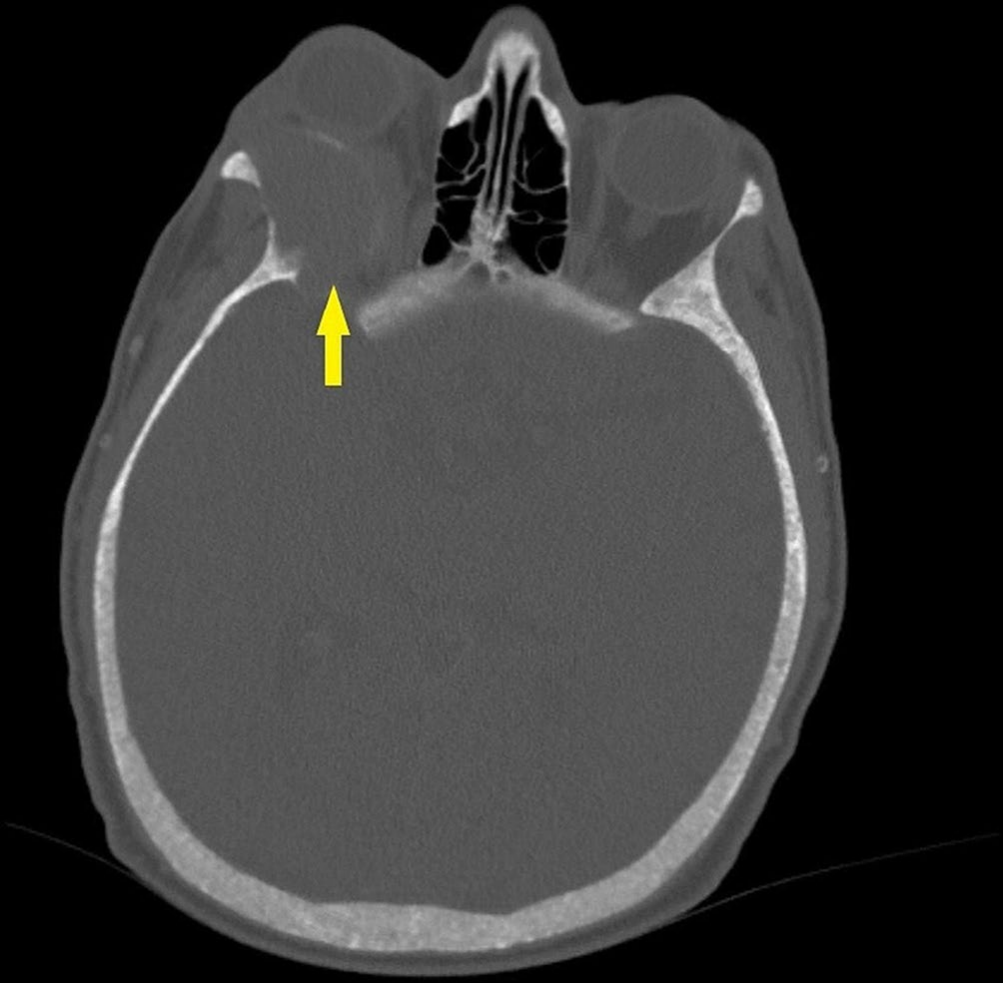
Cranial CT image. The arrow show tumoral lesion with peripheral linear calcification

**Fig. 3:**
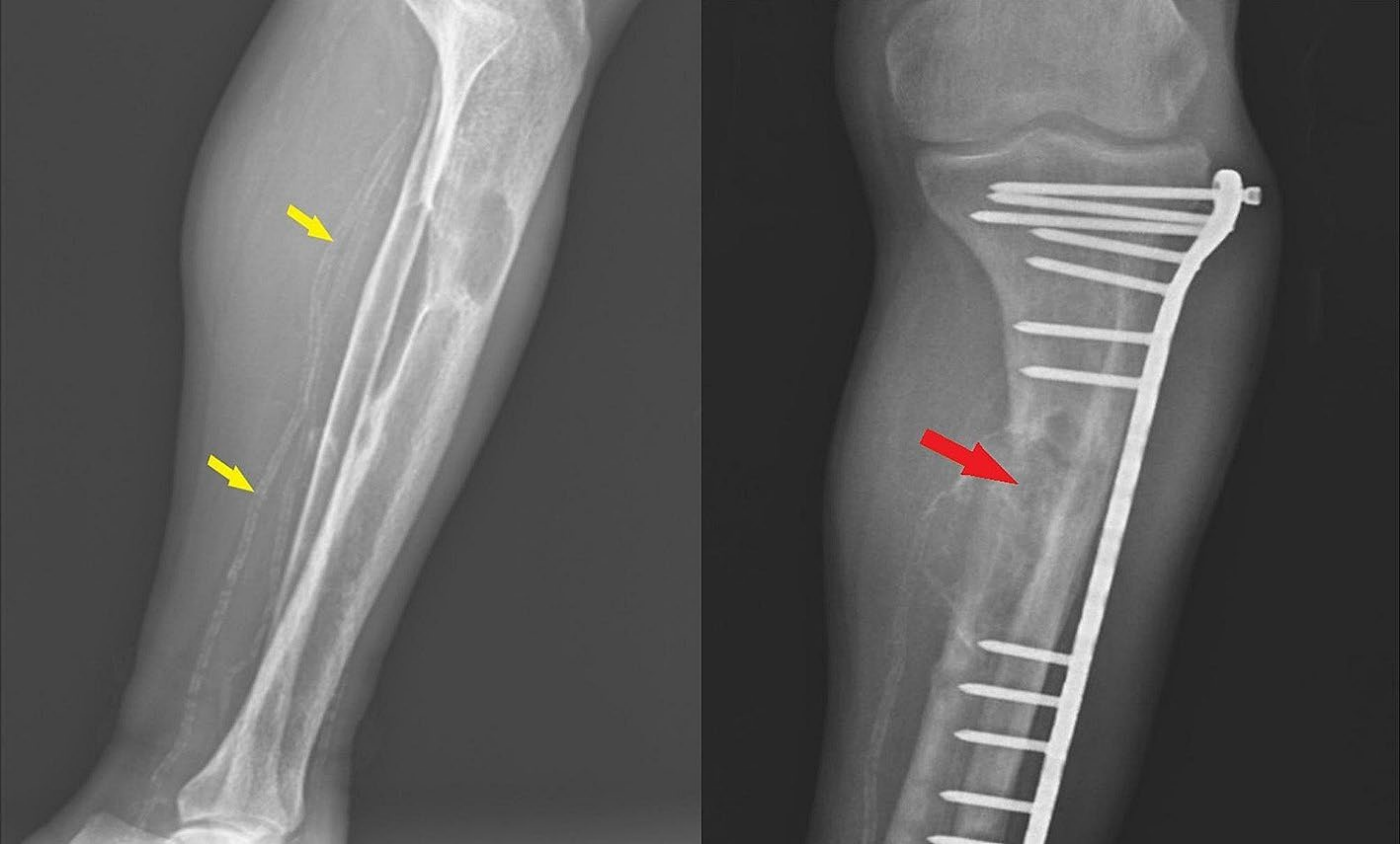
The X-ray radiography of the legs. The yellow arrows show vascular calcification in tibial artery and the red arrow shows osteitis fibrosa cystica on tibia

The immediate orbital and cranial imaging was performed by magnetic resonance imaging (MRI), and an extraconal mass lesion was detected in the right orbital posterior superolateral position. Mass lesion was exerting pressure on the orbital part of the frontal bone, the right optic nerve, and the superior rectus muscle. The intramedullary lesion measured 36*27*24 mm and exhibited diffuse homogeneous contrast enhancement in MRI. The tumoral lesion appeared slightly hyperintense on T1-weighted images and hypointense on T2-weighted images. The MRI sections are illustrated in Fig. 1. Afterward, the lesion was evaluated using computed tomography (CT). The CT scans revealed an expansile mass lesion with peripheral linear wall calcification at the right orbital posterolateral level and originating from the sphenoid wing (Fig. 2). The mass lesion caused erosion in adjacent bone structures, and was classified as a BT. The laboratory results were as follows: glucose: 92 mg/dL, blood urea nitrogen: 56 mg/dL, creatinine: 7.8 mg/dL, sodium: 137 mEq/L, potassium: 4.5 mEq/L, calcium: 9.7 mg/dL, phosphorus: 7.2 mg/dL, albumin: 3.8 g/dL, bilirubin: 0.4 mg/dL, alkaline phosphatase (ALP): 1427 IU/L, intact PTH: 4557 pg/mL, serum 25-OH vitamin D: 12 ng/mL, leukocyte: 9200 cell/µL, hemoglobin: 8.2 g/dL, platelet: 390 × 10³/µL. The patient underwent ileus surgery, parathyroidectomy, and leg fracture operation (Fig. 3) in medical history. However, the patient did not state that certain data about the parathyroidectomy operation however it was performed about 10 years ago. Furthermore, the patient had been complaining of muscle weakness and bone pain for years.

**Fig. 4:**
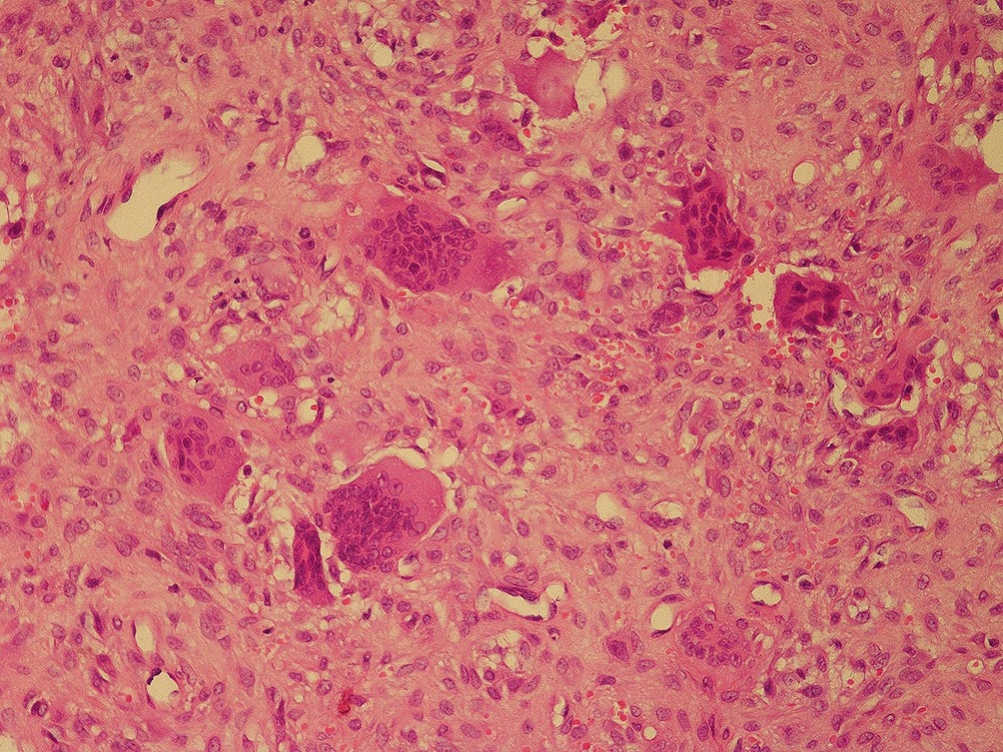
The image shows a pathological examination of the tumor in light microscopy (20xH&E)

The bone mass lesion was resected via craniotomy due to the compressive effect. During the operation, surgeons observed that the dura mater remained intact in the vicinity of the mass. There were no major complications noted during the postoperative period. Histopathological examination specimens supported the diagnosis of BT, the fibro-osseous lesion contained giant cells accompanied by bone resorption. Light microscopy revealed increased vascularity, fibroblastic proliferation, stroma containing extravasated erythrocytes, and osteoclast-type giant cells (Fig. 4).

**Fig. 5:**
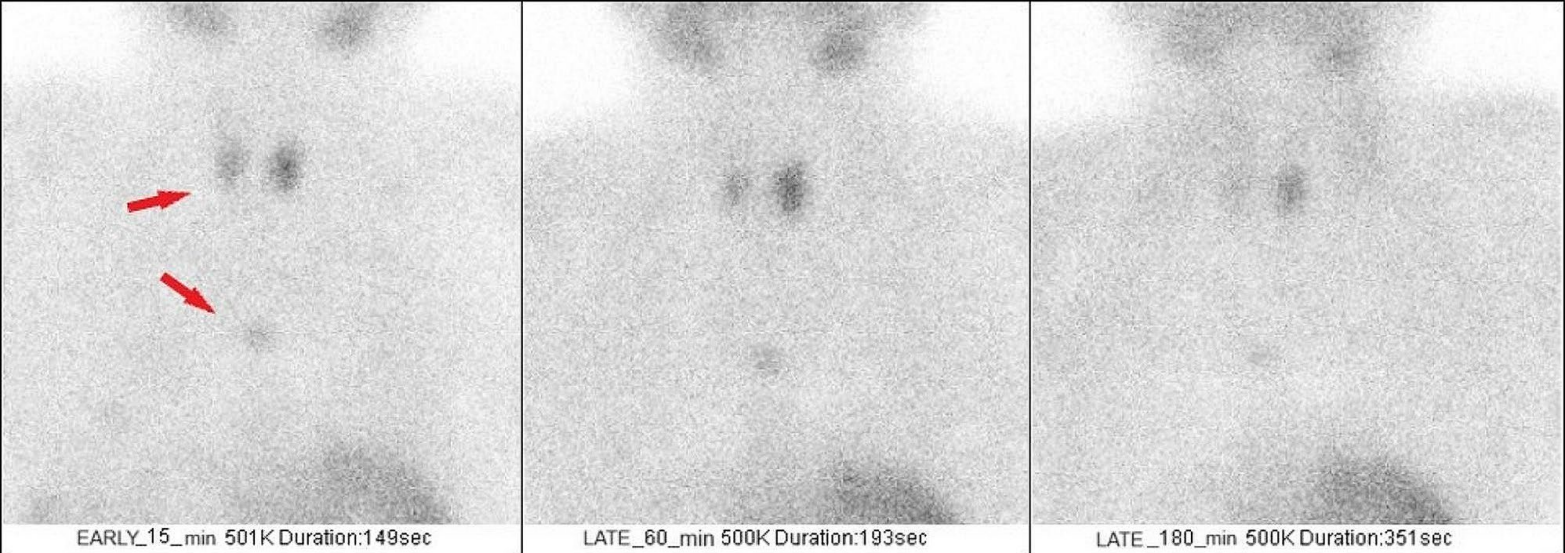
The images of parathyroid scintigraphy at different times.The arrows point to adenoma locations

The patient was evaluated for hyperparathyroidism and parathyroid scintigraphy with Tc-99 m methoxyisobutylisonitrile (MIBI) detected three distinct foci consistent with adenomas. The accumulations of Tc-99 m MIBI were sized at 25 × 14 mm inferior to the left thyroid lodge, 18 × 12 mm inferior to the right thyroid lobe, and 12 mm in the anterior mediastinal region, respectively. These findings were further confirmed by CT scan of the neck with contrast. The images of scintigraphy are shown in Fig. 5.

Parathyroidectomy was recommended because of refractory hyperparathyroidism. However, the patient refused the surgery due to concerns about debility and perioperative risks. Afterward, we retrospectively investigated dialysis inadequacy and reached out to the dialysis unit. Kt/V urea values had mostly been below 1.2 in the past year, and he had not been consistently taking his prescribed medications. Dialysis efficiency was enhanced through necessary adjustments and the average Kt/V urea increased to 1.7 over the next three months. The patient was receiving parenteral calcitriol and sevelamer treatment before the surgery. The patient previously declined oral cinacalcet because of gastrointestinal side-effects so parenteral etelcalcetide was initiated. He tolarated maximum 7.5 mg etelcalcetide, three times a week. Phosphorus levels decreased to target range (< 5.5 mg/dL) with lanthanum carbonate (3*1000 mg daily), calcium carbonate (3*2000 mg daily), and dietary phosphorus restriction. Paricalcitol, administered intravenously at a dose of 10 mcg three times a week, was introduced after phosphorus control. Unfortunately, the follow-up showed a decrease in the PTH level to a minimum of 1428 pg/mL. In the last laboratory assessment, serum calcium level was 10.4 mg/dL, PTH level was 2897 pg/mL and ALP was 1204 IU/L.

## Discussion and conclusions

Chronic kidney disease-mineral and bone disorder (CKD-MBD) presents significant challenges in the management of dialysis patients, encompassing various clinical disturbances. In this case, we focused on refractory hyperparathyroidism, a condition that posed severe clinical manifestations. This patient showcases the challenges in clinic management through all treatment steps and wretched complications of SHPT. The bone fractures, muscle weakness, bone pain, exophthalmos, cranial-located BT, and recurrent parathyroid adenomas were among these complications. Moreover, the patient had an extra parathyroid adenoma atypically located in the mediastinum. Therefore, comprehensive surgical intervention was required in this case. Thanks to effective treatment options in recent medical practice, the incidence of such severe cases has decreased.

The pathophysiology of CKD-MBD is complicated, involving intricate feedback loops between the kidneys, parathyroid glands, bones, intestine, and vasculature [7]. The heterogeneity of bone disorders in CKD-MBD leads to the identification of two major clinical entities: high-turnover and low-turnover (adynamic) bone disease. SHPT is an essential component of CKD-MBD and signifies high-turnover osteodystrophy. The term tertiary hyperparathyroidism is defined as permanent hyperparathyroidism, representing severe parathyroid hyperplasia and autonomous secretion of PTH independently of plasma calcium concentration in CKD.

Bone pain, osteoporosis, fractures, and BT formation are among the primary skeletal complications of SHPT. However, complications extend beyond the skeletal system in SHPT. Persistent elevation of the calcium-phosphate product exacerbates extraskeletal calcifications, which can occur in various sites, including the lungs, myocardium, and periarticular areas. Pruritus, calciphylaxis, neuropsychiatric symptoms, calcific uremic arteriolopathy, vascular calcifications, and soft-tissue calcifications might occur. Furthermore, SHPT is associated with elevated cardiovascular risk which is attributed in part to excessive vascular calcification. Additionally, SHPT causes a poor response to erythropoiesis-stimulating agent therapy and growth retardation in pediatric patients [8].

The classical manifestation of hyperparathyroidism is OFC, however, BT represents a severe form of OFC in prolonged hyperparathyroidism. BT are benign, rapidly growing, fibrotic, cystic bone lesions [9]. Despite BT is benign bone lesions, occasionally presented on imaging as an aggressive bone tumor.Lesions of OFC and BT can affect any bone, but commonly are located in the ribs, clavicles, pelvis, spine, tibia, humerus, and skull [10].

Atypical locations and diverse clinical presentations of BT have been documented in the literature. The clinical significance of BT depends on its potential to compress various organs. For instance, spinal cord compression due to BT, though rare, has been observed on multiple occasions [11]. While there is no specific treatment protocol for BT, surgical resection becomes necessary in cases where complications arise. BT serves as an indicator of an aggressive SHPT course.Unusual locations for BT, including the forearm, foot, jaw, mandible, sphenoid sinus, and calcaneus, have been reported.Orbital bones are exceptionally rare reported as BT location [12, 13]. Exophthalmos and visual impairment are the most common complaints among patients with orbital BT. In reported cases, early surgical intervention was undertaken due to the abrupt onset of symptoms, leading to resolution of visual damage in almost all patients during the postoperative period.

It is noteworthy that the majority of reported cases involving orbital BT had pre-existing CKD diagnosis, and most of these patients had ongoing dialysis therapy. Gonzalez-Martínez et al. reported a 30-year-old woman with proptosis who had no chronic disease before, and orbital BT was detected on imaging. As distinct from, kidney dysfunction was defined during perioperative period. The determination of kidney disease through orbital BT was very interesting in this case [14].

SHPT significantly impacts the mortality rate in dialysis patients, underscoring the necessity for effective control. The Kidney Disease Improving Global Outcomes (KDIGO) guidelines recommend maintaining PTH levels within 2 to 9 times the upper normal limit for the assay as the target range [15].Managing SHP begins with addressing hyperphosphatemia, with the goal of lowering elevated phosphate levels towards the normal range. The Kidney Disease Outcomes Quality Initiative (KDOQI) guidelines advise maintaining serum phosphate levels between 3.5 and 5.5 mg/dL. Phosphate-lowering therapies encompass dietary phosphate restriction, oral phosphate binders, and efficient dialysis. Ensuring normocalcemia is another crucial step in SHPT management. Calcitriol and synthetic vitamin D analogs can be employed to reduce PTH levels [16].In this case, paricalcitol was utilized afterward phosphate control. Calcimimetic agents operate by increasing the sensitivity of the calcium-sensing receptor (CaSR) in the parathyroid glands, thereby reducing PTH levels. Cinacalcet and etelcalcetide are widely available calcimimetic agents. Studies have demonstrated that cinacalcet therapy reduces the necessity for parathyroidectomy in dialysis patients [17].

Parathyroid surgery is recommended for patients with severe hyperparathyroidism that cannot be effectively controlled through medical means. Parathyroidectomy proves to be an efficacious therapy for refractory hyperparathyroidism and also contributes to the improvement of renal osteodystrophy [18]. Several indications may necessitate parathyroidectomy, including hypercalcemia, bone pain, refractory pruritus, unexplained myopathy, and calciphylaxis. For dialysis patients with PTH levels persistently exceeding 1000 pg/mL, even in the absence of obvious associated clinical symptoms, and who do not respond to medical therapies, referral for parathyroid surgery is recommended [19].

Hypocalcemia is a common issue following parathyroidectomy. The severity of hypocalcemia after surgery is directly related to the duration and extent of parathyroid-mediated high-turnover bone disease. Prolonged and severe postoperative hypocalcemia is known as hungry bone syndrome, andrefers to the hyperdynamic reabsorption of calcium into bones following parathyroidectomy [20]. There is a high risk of recurrent SHPT after a successful parathyroidectomy, with rates as high as 80% in dialysis patients. Consequently, some ESKD patients require repeated parathyroid surgeries for refractory disease [21].

In conclusion, we attempted to summarize the approach of SHPT via this patient. Management of patients with ESKD could present challenging and astonishing clinical scenariosto physicians. These patients exhibit numerous complications related to dialysis procedures or uremia. This unusual case emphasizes the importance of vigilance among all physicians, not solely nephrologists, especially concerning dialysis patients.

## Data Availability

Further clinical data and images of this case are available from the corresponding author upon reasonable request.

## Abbreviations

ALP: alkaline phosphatase
BT: Brown tumors
CaSR: Calcium-sensing receptor
CKD: Chronic kidney disease
CKD-MBD: Chronic kidney disease-mineral and bone disorder
CT: Computed tomography
FGF-23: Fibroblast growth factor 23
ESKD: End-stage kidney disease
HD: Hemodialysis
KDIGO: Kidney Disease Improving Global Outcomes
KDOQI: Kidney Disease Outcomes Quality Initiative
MRI: Magnetic resonance imaging
MIBI: Methoxyisobutylisonitrile
OFC: Osteitis fibrosa cystica
PTH: Parathyroid hormone
SHPT: Secondary hyperparathyroidism
